# Evaluation of failure modes and effect analysis for routine risk assessment of lung radiotherapy at a UK center

**DOI:** 10.1002/acm2.13238

**Published:** 2021-04-09

**Authors:** Martyn D. F. Gilmore, Carl G. Rowbottom

**Affiliations:** ^1^ Medical Physics Clatterbridge Cancer Centre NHS Foundation Trust Bebington Wirral UK

**Keywords:** FMEA, incident reporting, radiotherapy, risk analysis, risk assessment

## Abstract

**Purpose:**

Explore the feasibility of adopting failure modes and effects analysis (FMEA) for risk assessment of a high volume clinical service at a UK radiotherapy center. Compare hypothetical failure modes to locally reported incidents.

**Method:**

An FMEA for a lung radiotherapy service was conducted at a hospital that treats ~ 350 lung cancer patients annually with radical radiotherapy. A multidisciplinary team of seven people was identified including a nominated facilitator. A process map was agreed and failure modes identified and scored independently, final failure modes and scores were then agreed at a face‐to‐face meeting. Risk stratification methods were explored and staff effort recorded. Radiation incidents related to lung radiotherapy reported locally in a 2‐year period were analyzed to determine their relation to the identified failure modes. The final FMEA was therefore a combination of prospective evaluation and retrospective analysis from an incident learning system.

**Results:**

Thirty‐six failure modes were identified for the pre‐existing clinical service. The top failure modes varied according to the ranking method chosen. The process required 30 h of combined staff time. Over the 2‐year period chosen, 38 voluntarily reported incidents were identified as relating to lung radiotherapy. Of these, 13 were not predicted by the identified failure modes, with six relating to delays in the process, three issues with appointment times, one communication error, two instances of a failure to image, and one technical fault deemed unpredictable by the manufacturer. Four additional failure modes were added to the FMEA following the incident analysis.

**Conclusion:**

FMEA can be effectively applied to an established high volume service as a risk assessment method. Facilitation by an individual familiar with the FMEA process can reduce resource requirement. Prospective evaluation of risks should be combined with an incident reporting and learning system to produce a more comprehensive analysis of risk.

## 1 INTRODUCTION

Modern radiotherapy is recognized as a highly complex, multistep process delivered by a multidisciplinary team requiring numerous handovers.^1^ Although radiotherapy is widely considered a safe and effective treatment option for cancer patients, radiotherapy accidents can have severe consequences resulting in significant patient harm.^2^ In the last decade, there has been extensive work carried out to improve radiotherapy safety and risk assessment in the United Kingdom,^3^ Europe,^4^ and the United States.^5^ To ensure the safety of treatments is established and maintained, regular risk assessment forms a key aspect of the commissioning and review of treatment techniques^3^ and remains a legal requirement under UK law.^6^ More generally, the IAEA Basic Safety standard,^7^ from which most national safety standards are derived, also emphasizes the need to reduce radiological accidents and evaluate risks.

### 1.A Failure mode and effect analysis

There are a variety of tools available to facilitate risk assessment, with failure mode and effect analysis (FMEA) generating a significant level of interest as an appropriate tool for use in radiotherapy, most notably in the AAPM task group 100 report.^8^ In the literature, FMEA has been applied successfully to several complex radiotherapy modalities such as Radiosurgery and Stereotactic Body Radiotherapy.^9^, ^10^ The methodology advocated by Huq et al.^8^ starts with a process map of the steps associated with the application requiring analysis. An FMEA is then performed to assess the likelihood of failures during each step in the process and the potential impact of such a failure. For each potential failure mode, the associated risk is classified using three parameters, Severity, Occurrence (or frequency), and Detectability according to the scoring system proposed by TG‐100.^8^ For a center considering an FMEA‐based approach to risk assessment a number of challenges identified within the literature require some local adaptation and interpretation. They are discussed below.

### 1.B Identification of failure modes

Identifying potential errors and risks within a complex multistep process can be an exhaustive process and limited conclusions can be drawn from the available literature. Considering examples published on radiosurgery, the number of failure modes identified ranged from 86^11^ to 409.^12^ For more general radiotherapy, papers have been presented by several groups^13^, ^14^, ^15^ with failure modes identified ranging from 52 to 127 with a variety of treatment planning and delivery platforms, and variable discussion of the scope of FMEA (i.e., including acceptance/commissioning as well as routine use).

### 1.C Risk priority number

The risk priority number (RPN) is used to stratify failure modes by multiplying severity, occurrence, and detectability into a single number. The use of RPN is a recognized issue with the FMEA format^16^, ^17^ not least as the doubling of a number, for example, severity does not result in a doubling of RPN.^18^ With use of an in‐direct indicative score such as RPN, the method for stratifying risks and identifying those to focus intervention on is critical. Methodologies presented vary from the top 5%^19^ to all modes^20^ most likely reflecting the relative time and resources available between groups. Direct reliance on RPN for stratification is not recommended by Huq et al.^8^, with some manual interpretation of severity suggested. In addition, the scoring of severity, occurrence, and detectability, and subsequent RPN can produce considerable variation between individuals even with the use of a standard matrix.^21^ With multiple participants, a careful choice must be made between averaging of individual scores or group consensus scoring, with Ashley and Armitage (2010) recommending a consensus approach that allows for review and discussion of variation^22^

### 1.D Resource implications

Process mapping, identifying, and ranking failure modes and further intervention can require considerable resources.^17^ There is limited consensus or discussion of the resource implications for an individual center considering adopting FMEA risk assessment. For surface guided radiotherapy, identification of failure modes and validation of occurrence, severity, and detectability was estimated at 30 hours.^23^ For a general external beam process with support from a trained facilitator, total FMEA including analysis was estimated at 75 h.^15^ Three centers exploring FMEA for radiosurgery identified 104–135 failure modes but completed the process over a period of 2–6 months.^10^ The most extensive example of an FMEA might be that offered by Schuller et al.^12^ who identified a total of 409 failure modes, applied analysis to all modes and required an estimated total of 258 h, equivalent to 34 and a half working days. Variation and uncertainty in resource requirements could potentially act as a barrier to a center considering FMEA‐based risk assessment for new or existing services and prevent more widespread practical implementation.

### 1.E FMEA for lung VMAT

The aim of the work presented here is to evaluate the application of FMEA as a tool for prospective risk assessment within a UK hospital. The FMEA approach has been adopted locally as a methodology for documented radiation risk assessments within our center, as required under UK legislation.^6^ A specific indication, lung cancer, has been chosen as the focus due to its high throughput, universal application, and relative complexity due to motion management issues.

Lung cancer accounts for 13% of cancer diagnoses in the United Kingdom with 46,403 cases diagnosed in 2014.^24^ Noticeably, deaths attributed to lung cancer represent 22% of all cancer deaths, with 1‐year survival rates of 34% and 39% for men and women, respectively.^25^ At our center, approximately 350 patients per annum undergo radical radiotherapy for lung cancers using volumetric modulated arc therapy^26^ (VMAT), a technique by which radiation is delivered in either a single or multiple arcs with varying field aperture, dose rate, and rotation speed to produce a highly conformal dose distribution.

At our center, patients undergo 4D CT scans with motion management provided by Varian’s RGSC system (Varian, Palo Alto, USA). Planning is carried out using Eclipse (version 15.6) and delivered on True‐beam (version 2.5) with Aria as the record and verify system and IGRT provided by 4D and 3D CBCT. The patient pathway is paperless with individual tasks forming part of a Care Path, (Varian, Palo Alto, USA) effectively a standardized electronic process map of the pretreatment workflow for each individual patient. Planning is carried out with the aid of automatic scripting and questionnaires. Our center uses the incident reporting system, Datix (RLDatix, London, UK) for staff to raise incidents and near misses, with incidents reported voluntarily to the NHS England and Wales National Reporting and Learning System (NRLS) at NHS Improvement^27^ as recommended by Towards Safer Radiotherapy.^3^ Incidents reported are assigned a level according to the severity classification scale.^3^

The work presented here, outlines the application of FMEA to lung radiotherapy within our center, and includes discussion of current stratification methods and rankings, as well as comparison to incident reports as a measure of efficacy of the process. The emphasis of the paper is on the process of risk assessment and stratification rather than on resolution of weaknesses identified by failure modes.

## 2 MATERIALS AND METHODS

The FMEA exercise described herein was undertaken during a 3‐month period at a UK NHS hospital. A multidisciplinary team was recruited for the exercise, consisting of two oncologists, two physicists, and three radiographers. To expedite the process, the lead author (a physicist) acted as facilitator and produced a process map and initial failure mode list based on historical risk assessments and the authors own understanding of the process. As the FMEA was for a pre‐existing service, failure modes related to the commissioning process of the technique and equipment were omitted. Members of the group then individually scored the provided modes using the TG‐100 scoring matrix and were asked to identify any additional failure modes. A face‐to‐face multidisciplinary team (MDT) meeting was then held to discuss the additional modes and review failure modes with a high variation (>5) in either severity, occurrence, or detectability score between MDT members. After consensus was achieved, to determine the validity of pre‐existing US originating taxonomy to UK practice, local failure modes were assigned to the generic steps identified by Ford et al.^28^ with the local expected causes linked to the associated coded causality.^28^

To illustrate the high‐risk process steps, the final FMEA modes were transcribed onto the process map. To determine the resource requirements of the FMEA process, the facilitator and all participants were asked to record time spent on the FMEA and the face‐to‐face meeting was timed.

To evaluate the usefulness of scoring systems the final local FMEA scores were stratified according to RPN number and a novel three‐digit code system that uses severity, occurrence, and detectability to produce a three‐digit number: S, O, D, that provides direct information on each category. To facilitate a three‐digit code, the 1–10 system^8^ was adjusted to 0–9 by subtracting 1 from all individual severity, occurrence, and detectability scores. The three‐digit code system provides greater flexibility to consider risks in terms severity, occurrence, and detectability independently, and mitigates the limitation of RPN that different combinations of S, O, and D can produce exactly the same value of RPN despite potentially having very different risk implications.^29^

Finally, to determine the efficacy of the FMEA process, the centers incident reporting system, Datix, was interrogated to find all reported incidents involving patients undergoing VMAT Lung radiotherapy since its implementation in April 2017. These results were then vetted for relevance to the radiotherapy planning process, compared to the identified failure modes and added to the process map.

## 3 RESULTS

For the FMEA, 34 modes were identified by the facilitator and sent out to each individual for scoring. From the original 34, 17 (50%) had a variation in ranking of either severity, occurrence, or detectability greater than 5. From the MDT meeting, these were discussed in detail and final scores taken by consensus. In addition, two modes were removed and a further four added. The final number of failure modes identified was 36. The top 10 Failure modes ranked separately by RPN, and S,O,D are found in Table 1 and Table 2, respectively.

**Table 1:** Failure modes ranked by RPN.

Function or Process Step (local)	Process map (Ford et al.)		Failure Type	Potential effect	SEV (1‐10)	Potential Causes	Causality Coding (Ford et al.)	OCC (1‐10)	Preventative measures	Detection Mode	DET (1‐10)	RPN	RPN S,O,D
Coaching decision		2.2 Imaging	Coaching chosen over free‐breathing when not needed	Target motion increased ‐ increases treatment volume	5.0	Human error	3c poor judgment	8.0	Training	Large volume may be rejected by Oncologist/violate dose constraints	10.0	400	479
Dose check	4.2 Independent Dose check		Dose check fail overridden	Error in dosimetry	9.0	Human error	6ciii Failure to select the correct rule	4.0	Clear flow charts on process for training,	None	10.0	360	839
Plan check	4.1 Physicist plan review		Suboptimal plan check	Errors in plan not found	4.0	Human error	6a Failure to detect a developing problem	8.0	Script and questionnaire prompts, previous controls at early stages	Limited checks by dose calculation	10.0	320	379
IGRT	5.1 Image guided verification		Error in match < 5 mm	Positional error, one fraction	4.0	Human/procedural error	3c poor judgment	9.0	training, two radiographers present for match	Large magnitude moves require TEP, bone and PTV match carried out	7.0	252	386
Gated Tx prep	3.12 Setup for IGRT		Isocenter wrong and/or AIP wrong on imaging plan	IGRT CT error	6.0	Human error	6dv failure to develop an effective plan	4.0	Original plan locked, training	Comparison to treatment plan	8.0	192	537
Plan production	3.7 Dose distribution optimization		Planner produces poor quality plan	Under/over‐treatment of target/OARs	7.0	Human error	6dv failure to develop an effective plan	5.0	Dose constraints in template, training	Plan checker reviews plan quality, Oncologist decides on acceptability	5.0	175	644
Clinician outlining and prescribing	3.2 Delineation of target		Oncologist outlines target incorrectly	Under/over‐treatment of target/OARs	8.0	Poor scan quality, no access to previous scans, human error	3c poor judgment	3.0	Training, experience, confirmatory radiologist reports of previous scans	Macro level inspection by planner/checker	5.0	120	724
IGRT	5.1 Image guided verification		Error in match > 5 mm	Positional error, one fraction	5.0	Human/procedural error	3c poor judgment	3.0	Training, two radiographers present for match	Large magnitude moves require TEP, bone and PTV match carried out	7.0	105	426
Radical RT actioned	1.1 Decision to treat		Oncologist prescribes suboptimal dose/fractionation	Under/over‐treatment of target/OARs	8.0	Incomplete patient information, human error	3c poor judgment	3.0	Clearly defined protocol, training and experience	planner/checker check protocol but cannot advise on appropriateness	4.0	96	723
Patient not suitable for radiotherapy	1.1 Decision to treat		Patient does not fulfil eligibility/referral criteria	Inappropriate CT and (potentially) treatment	10.0	Incomplete information provided by referrer, unknown additional comorbidities	3c poor judgment	3.0	Oncologist consultation, EAS prompts	Consultant outlining, review of referral by CT staff. Inspection of notes by radiotherapy planner	3.0	90	922

**Table 2:** S,O,D ranked failure modes.

Function or Process Step (local)	Process map (Ford et al.)		Failure Type	Potential effect	SEV (1‐10)	Potential Causes	Causality Coding (Ford et al.)	OCC (1‐10)	Preventative measures	Detection Mode	DET (1‐10)	RPN	RPN S,O,D
Patient selection		1.1 Decision to treat	Patient does not fulfil eligibility/referral criteria	Inappropriate CT and (potentially) treatment	10.0	Incomplete information provided by referrer, unknown additional comorbidities	3c poor judgment	3.0	Oncologist consultation, EAS prompts	Consultant outlining, review of referral by CT staff. Inspection of notes by radiotherapy planner	3.0	90	922
CT scan	2.1 Verification of patient ID		Patient does not fulfil eligibility/referral criteria	Inappropriate CT and (potentially) treatment	10.0	Incomplete information provided by referrer, unknown additional comorbidities	6e Failure to execute the planned action	3.0	Oncologist consultation, EAS prompts	Consultant outlining, review of referral by CT staff. Inspection of notes by radiotherapy planner	3.0	90	922
Clinician Outlining	3.2 Delineation of target(s)		Wrong site outlined	Complete erroneous plan	10.0	Human error, limited information	3c poor judgment	2.0	Radiologist reports, training and experience	plan checker looks for independent confirmation of laterality	2.0	40	911
1^st^ fraction	5.1 Verification of patient ID		wrong patient	complete wrong treatment	10.0	human error	6e failure to execute the planned action	2.0	Patient ID check	IGRT	2.0	40	911
Dose check	4.2 Independent Dose Calculation		Dose check fail overridden	Error in dosimetry	9.0	Human error	6ciii Failure to select the correct rule	4.0	Clear flow charts on process for training	None	10.0	360	839
Treatment prep	4.6 Verification of parameters		Plan changed from prescribed/checked	Error in treatment delivery	9.0	Human/procedural error	6a failure to detect a developing problem	3.0	Plans locked after planning	Time stamps on plan check comments verified on first fraction	2.0	45	821
Import	2.1 Transfer of images to treatment planning system		Wrong scan/incomplete scan imported	Error in plan	9.0	Human error, import failure	6e failure to execute the planned action	2.0	Aria data integrity check	Planner checks image parameters	2.0	36	811
Dose check	4.2 Independent Dose Calculation		Dose check process/calculation error	Error in dosimetry	9.0	Software error, human error	6e failure to execute the planned action/2bv software operation failure	1.0	Automated dose check process, version controlled and CE marked software	none	10.0	90	809
Incorrect target outlining	3.2 Delineation of target(s)		Oncologist outlines target incorrectly	Under/over‐treatment of target/OARs	8.0	Poor scan quality, no access to previous scans, human error	3c poor judgment	3.0	training, experience, confirmatory radiologist reports of previous scans	Macro level inspection by planner/checker	5.0	120	724
Sub‐optimal prescription chosen	1.1 Decision to treat		Oncologist prescribes suboptimal dose/fractionation	Under/over‐treatment of target/OARs	8.0	Incomplete patient information, human error	3c poor judgment	3.0	Clearly defined protocol, training and experience	Planner/checker check protocol but cannot advise on appropriateness	4.0	96	723

### 3.A Incident reports

Between April 1, 2017 and March 31, 2019, 750 patients were treated with VMAT for lung cancer at our institution. From interrogation of the local incidents database, 38 incidents were identified as relating to lung radiotherapy from this period. Seventeen (45%) were classed according to the classification scale^3^ as level 5 (non‐conformance), 17 (45%) as level 4 (near miss) and 4 (11 %) as level 3 (Minor radiation incident). The low rate of level 3 incidents highlights the mature development of robust risk mitigations in place for the existing clinical service. For each, the incident report was reviewed to determine during which process step the failure occurred and whether the incident was predicted by a specific failure mode. Of the 38 incidents, 13 (34 %) were not attributable to failure modes identified by the FMEA MDT meeting. Of the 13 not predicted by failure modes, six (46%) were delays in the process and three (23 %) were issues with appointment time scheduling. The remaining four were:

- A communication error whereby the patient was not told of a change in treatment intent
- A technical fault in plan generation that, after investigation and discussion with manufacturer was considered rare and unpredictable and resulted in an undeliverable plan
- Two instances of a failure to image when required on a weekly basis

### 3.B Lung VMAT radiotherapy process map

**Fig. 1:**
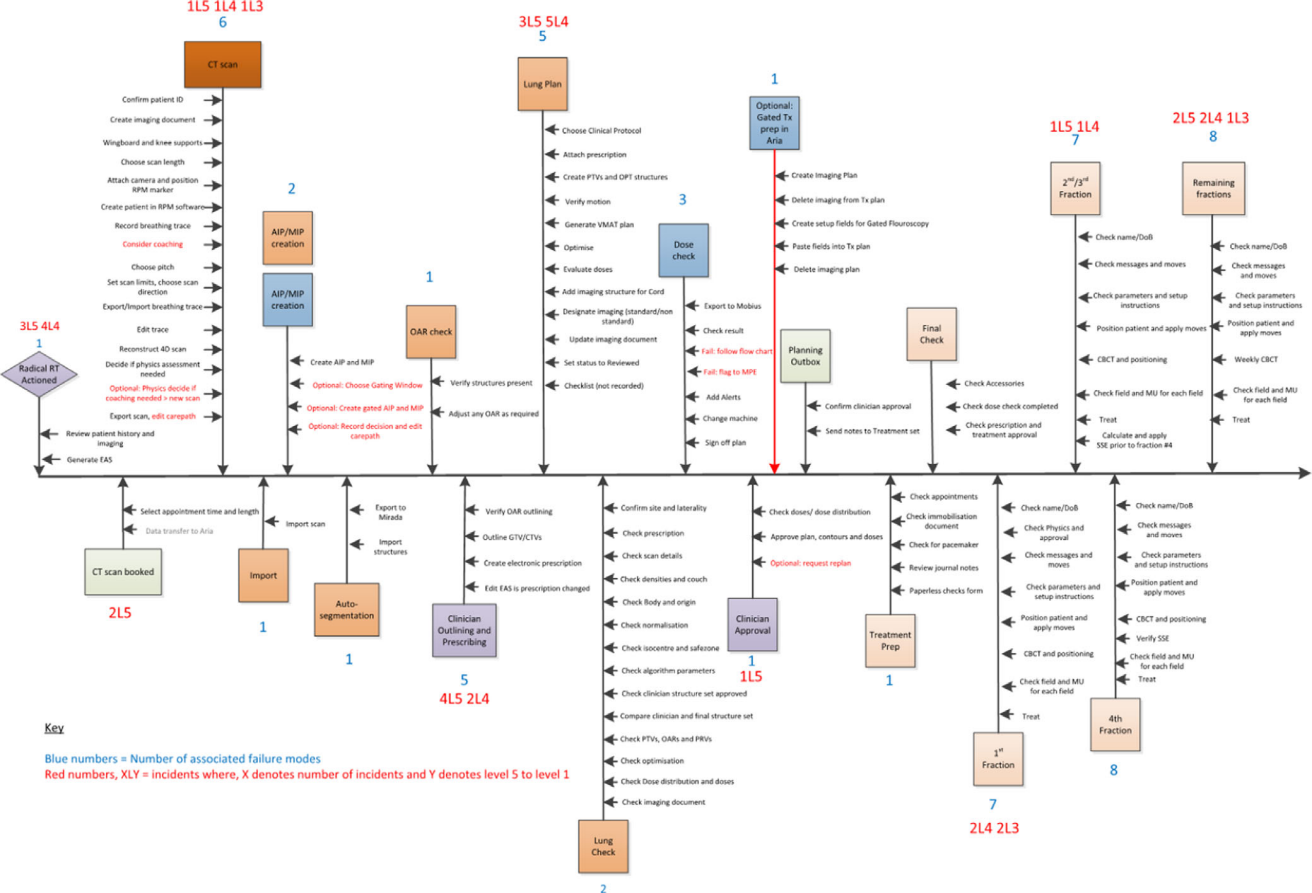
Lung Radiotherapy process map including failure modes and reported incidents.

A high level process map was created to reflect the local Care Path with 20 steps from the creation of an electronic action sheet (EAS) to the end of treatment. Failure modes identified and attributable reported incidents were mapped to the relevant process steps (Fig. 1).

### 3.C Resource requirements

The total staff time invested in this project was 29.5 h including 14 h (47%) from the facilitator. The MDT members took on average 1.5 h to review the FMEA and process map (range 0.75–2) and the consensus review meeting took 2 hours.

## 4 DISCUSSION

The work presented here demonstrates the practical application of the FMEA approach to a high volume radiotherapy service at a large UK hospital. By using a facilitated approach similar to that of Ford et al.,^15^ an MDT group was able to produce a full FMEA and process map for a pre‐existing VMAT lung service. The process highlighted the key control measures of plan checking, pretreatment checks, and IGRT at first fraction. The 36 failure modes identified represent a relatively low level of resolution and are not exhaustive. As an example, a planner producing an undeliverable plan was recorded as a single failure mode, which could have been caused by a number of potential sources from incorrect isocenter position to over modulation. This highlights a key difficulty of risk assessments of complex processes, of which FMEA is one approach, whereby a large number of discrete errors in a specific process step can produce subtly different failure modes. Use of the FMEA process, from the local experience, becomes an exercise in balancing the time taken with the resolution of the analysis. For the presented example as an established service, each step on the patient Care Path already has existing control measures designed to identify potential failures. For example, plan production at our center incorporates a combination of Eclipse scripting (Varian, Palo Alto, USA) and electronic questionnaires based on the potential errors specific to the planning process. The FMEA for the entire lung process was conducted with this in mind.

When carrying out FMEA for a new service, with no such work in place, the granularity of the FMEA should be increased. When considering the number of failure modes to be expected, it is clear from the literature that this can vary considerably for similar treatment techniques. Numerous examples exist for radiosurgery, with Masini et al.^30^ identifying 116 failure modes, Younge et al.^19^ finding 99 failure modes, Schuller et al.^12^ stating 409 failure modes. In 2016, Teixeira et al.^10^ carried out independent FMEA for radiosurgery at three separate centers finding 135, 104, and 131 at each center, respectively. A key difficulty when looking for guidance within the literature is no unified approach to reporting. Limited information is available on the software systems and versions, treatment and imaging equipment including record and verify systems. Also unclear is whether specific aspects such as treatment planning system and machine commissioning are included, or assumed correct at the time of the FMEA. To improve the standardization of reporting FMEA application the authors would recommend reporting the equipment and systems used to deliver the service. In the example provided here the authors have attempted to clarify the focus of the FMEA and the equipment involved.

The importance of face‐to‐face discussion of the FMEA should be emphasized, as initial ranking by individuals results in 17 modes (50% of the original 34) with high variability (>5) in a least one of severity, occurrence, or detectability, with 10 (29%) having a variation of 7 or greater. From discussion, it was clear that failure modes are often multifaceted and discrepancy can arise if considering either a more severe but relatively unlikely error, or a more frequent but less severe alternative. Such discrepancy can either be resolved by agreeing to a specific circumstance, or by considering subdividing the mode particularly if the control measures change with the magnitude of error. An example would be accidental density override by a planner — here the error can be relatively small, if for example gas is not overridden, or large, such as if a lung is erroneously overridden as tissue.

### 4.A Translation of FMEA to UK practice

There are several discrepancies between typical US and UK practice, most notably in the difference between UK radiographers and US dosimetrists and the limited availability of clinicians to attend at treatment in the United Kingdom. Comparison of local practice for VMAT lung to the generic process map provided by Ford et al.^28^ was, however, straightforward with the majority of failure modes fitting within the process steps and causality coding provided. Some differences arise when considering the limited physicist involvement in the local VMAT lung service where radiographers carry out plan checks. The use of causality coding was found to be particularly useful as it provided a better resolution to user related errors than simply a general classification of “operator error.”

### 4.B Stratification and RPN

The RPN is a multiplication of the three S, O, and D indices designed to facilitate prioritization of failure modes. In its report, TG 100^8^ highlights two key issues with RPN, first that the severity of errors can often be inversely proportional to their likelihood and detectability and second that RPN ranking alone can risk loss of focus on the most hazardous steps.

When adopting streamlined FMEA for regular risk assessment of existing services rather than the commissioning of new techniques, the authors recommend avoiding stratification with RPN and replacing with the concept of a more directly informative 3‐digit code. For this, the FMEA indices scale is adjusted to be 0–9 from 1–10 and the combination of severity, occurrence, and detectability listed as S,O,D. This would give an S,O,D code of 911 for the wrong patient being called for treatment for example. Ranking by S,O,D produced a top 10 failure modes ranked by severity, but also, allowed for clear identification of a failure mode according to its severity, occurrence, and detectability.

The differences between the top 10 failure modes identified by RPN and S,O,D are shown in Table 1 and Table 2. Of the top 10 modes ranked according to S,O,D, only four appear in the top 10 ranked by RPN, and only five in the top 50 %. The modes ranked outside the top 50 % by RPN include those considered locally to be “never events” analogous to those outlined in the NHS England Never Events list^31^ based on their severe consequence, wholly preventable nature and likelihood of qualifying as reportable incidents under UK legislation^32^ These include wrong patient or wrong site related errors which given their rarity have a low natural likelihood and detectability. For an example failure mode, where the wrong patient is called for treatment, the MDT group ranked severity as 10, as this is an unintended irradiation of a patient. Occurrence was considered to be low, but not impossible at two, and detectable, with a score of two, given the local requirements for two stage identification and the use of IGRT. This produces a relatively low RPN of 40, ranked 31st of 36.

For a high volume service, even a low likelihood can produce a significant number of events. Under RPN ranking alone, there is risk for such catastrophic errors to be under‐emphasized and not revisited as part of future updates to the risk assessment. Conversely, the risk‐grading matrix proposed by Huq et al.^8^ can overemphasize low severity, high frequency failure modes. In the example of a suboptimal IGRT match, where the radiographers position the patient < 5 mm away from the intended isocenter, this will produce a minor dosimetric error (rank 4 severity) but is likely to occur relatively often, between 2 and 5% of the time (occurrence rank 9). A 10% risk of the failure going undetected (detectability rank 7) produces an overall RPN of 105, the fourth highest by RPN ranking. A IGRT match error of a greater magnitude (e.g., >5 mm), was considered locally as a separate failure mode, with a severity of rank 5, likelihood of rank 3, and detectability of rank 5, ranked lower by RPN (ninth) despite a higher severity. The failure mode was divided in this way as the associated controls at our center differ with magnitude, with errors larger than 5 mm requiring an independent expert practitioner to review the IGRT match. Splitting failure modes in this way can ensure different outcomes are considered, but will also increase the total number of modes requiring analysis.

When considering actions, ranking failure modes according to each indices can aid stratification, but for the implementation of FMEA for regular risk assessment of services, each mode should be reviewed in case of any opportunity for improvement.

### 4.C Incident reporting and FMEA

Of the 25 incident reports attributable to identified failure modes, 18 (72%) resulted from the five failure modes listed in Table 3.

**Table 3:** Five most commonly occurring Failure modes according to locally reported incidents.

Function or Process Step	Failure Type	Potential Impact	SEV (1‐10)	Potential Causes	OCC (1‐10)	Detection Mode		DET (1‐10)	Number of reported incidents
Patient not suitable for radiotherapy	Patient does not fulfil eligibility/referral criteria	Inappropriate CT and (potentially) treatment	10.0	Incomplete information provided by referrer, unknown additional comorbidities	3.0	Oncologist consultation, EAS prompts	Consultant outlining, review of referral by CT staff. Inspection of notes by radiotherapy planner	3.0	4
Sub‐optimal prescription chosen	Oncologist prescribes suboptimal dose/fractionation	Under/over‐treatment of target/OARs	8.0	Incomplete patient information, human error	3.0	Clearly defined protocol, training and experience	Planner/checker check protocol but cannot advise on appropriateness	4.0	4
Overly modulated or geometrically impossible plan produced	Planner produces undeliverable plan	Error in dosimetry	7.0	Human error, unrealistic oncologist request	4.0	Aria has limits for MLC, training, MU/cGy values	Plan checker reviews plan deliverability. Mobius independent dose calculation	2.0	3
Planner states imaging tolerances for patient based on cord dose	Planner states incorrect tolerances or fails to designate a bone match when needed	Poor setup accuracy resulting in under/over treatment of target/OARs	7.0	Human error	3.0	Clear protocol for choice	Plan checker verifies imaging tolerances	3.0	4
Wrong plan	Plan changed from prescribed/checked	Error in treatment delivery	9.0	Human/procedural error	3.0	Plans locked after planning	Time stamps on plan check comments verified on 1st fraction	2.0	3

Of the reported incidents, 2/38 (5%) were not detected by the expected control measure. In both cases, this was a failure of the radiographer checking the plan to spot errors in the immobilization information that was discovered on first fraction due to the use of IGRT as a safety barrier. No incidents were reported as resulting in any harm or potential harm to the patient. From this relatively low number of incidents, it can be argued that the frequencies stated within the FMEA exercise would appear to be higher than reality. There are, however, caveats to incident reporting systems that must be considered. The incidents reported cannot include all potential near misses and errors that are undetected and thus unreported. The system relies on voluntary reporting and incidents that occur and subsequently rectified may not be included. Physicists and radiographers reported all 38 incidents with no incidents directly reported by a clinician. There is therefore a risk of bias in the type of incidents reported due to a nonuniform culture of incident reporting between professions.

From the incident analysis, it is clear that the local FMEA did not include delays and communication errors as failure modes. Although errors in the imaging process were included, a failure to image when required was not an explicit failure mode. Local discussions focused primarily on erroneous actions, rather than nonactions and delays. The lack of inclusion of missed imaging as a failure mode is perhaps a direct oversight, but failure to include delays and communication errors may be in part due to a focus on scoring severity, occurrence, and detectability. Delays often do not contribute quantifiable consequences despite their potential effect on patient experience. The communication error, whereby a patient was not informed of a change in treatment, again does not produce a quantifiable consequence in terms of treatment outcome but has a significant effect on patient experience. Given FMEA has been developed and used in industry, an unexpected consequence may be a focus on measureable outcomes and, in healthcare, physical harm.^17^ Despite the importance of patient experience and the potential contribution to staff stress from delays and communication errors,^33^ it can be shown from this single center experience that the FMEA process can potentially lead to omission of these risks. A potential modification to the FMEA process could be to formally consider, once the process map has been established, what controls are in place to ensure the process step is performed. This would naturally result in a discussion of the consequences of omission, delay, and effect on patient experience. Even with the adoption of this modification a more holistic approach, utilizing a range of quality measures including patient outcomes, risk assessments, incidents, near misses, and patient experience measures, would be recommended to ensure sustained quality improvement.

An additional incident, a technical fault within the record and verify system, was investigated by the manufacturer and classified as rare and unpredictable. Although this error did not produce a physical effect, as the plan was undeliverable, the incident highlights the impossibility of a fully exhaustive proactive risk assessment. In reality, there is an unpredictable element that relies on nonspecific control measures and the competency and skill of staff involved that may not be explicitly included within the FMEA. This highlights the importance of combining FMEA risk assessment with a local incident reporting and learning system.

Considering the incidents reported locally, four additional failure modes were added to the risk assessment, listed in Table 4.

**Table 4:** Additional failure modes identified from the analysis of locally reported incident.

Function or Process Step (local)	Process map (Ford et al.)	Failure Type	Potential effect	SEV (1‐10)	Potential Causes	Causality Coding (Ford et al.)	OCC (1‐10)	Preventative measures	Detection Mode	DET (1‐10)	RPN	RPN S,O,D
Clinician Outlining	3.2 Delineation of target(s)	Clinician not available to outline	Delayed treatment	3.0	Clinician availability/cover not arranged	1ai inadequate human resources	4.0	Oncologist arranges cover, job plan review	Patient workload reviewed by planning to detect delays	1.0	12	230
Delay to plan or check	3 treatment planning	Plan and/or check delayed	Delayed treatment	2.0	Incomplete information of clinician intent/staff not available/Care Path error	1ai Inadequate human resources 6e Failure to execute the planned action	2.0	Care Path prioritizes overdue plans	Script checks for unattached patients, planning review list	1.0	4	110
IGRT	5.1 Image guided verification	Images not acquired when requested	Positional error	4.0	Human error, in correct information	3c poor judgment 6e Failure to execute the planned action	4.0	Images scheduled for correct dates, immobilization document	Weekly review of imaging by treatment team	4.0	64	333
Plan corruption	4.1 other	Software bug prevents use of plan	Delayed treatment	2.0	Software error	2bv software failure	1.0	None	Plan will not deliver	1.0	2	100

The failure of the local FMEA to anticipate 13 of the 38 identified incidents is comparable to the experience of Yang et al.,^34^ whose validation for an FMEA for SBRT found 13 of 33 incidents were not identified by an FMEA. Similarly to the finding of Yang et al. the incidents that were not anticipated by FMEA were of low average severity, occurrence, and detectability (< 3 in all cases).

The addition of the analysis of incident reporting is clearly of benefit during FMEA if available.^35^ Voluntary anonymized reporting of incidents using national or international systems may provide useful data for centers developing new services already established elsewhere. Local incident reporting and learning systems are a crucial tool for monitoring safety following the introduction of new risk mitigations resulting from FMEA risk assessment.

### 4.D High‐risk process steps

Within the process map in Fig 1, the number of modes associated can be used to highlight particularly complex process steps. Steps with greater than three failure modes are CT scanning, outlining, planning, and first treatment. From the incident reporting analysis, the majority of actual incidents occurred at: outlining (6), planning (8), the decision to treat (7), and first treatment (5). Using the number of associated failure modes to highlight key process steps would have not included the decision to treat as a high‐risk step for further detailed investigation.

The decision to treat process step represents a stage in the local patient pathway whereby an electronic action sheet (EAS) is produced by the clinician to specify the intended dose and fractionation, state the target laterality, and highlight any previous radiotherapy. A number of incidents occurred at this stage but in the local FMEA only two failure modes were identified for the EAS process step. The first, a patient incorrectly prescribed radiotherapy and the second a patient prescribed a suboptimal combination of dose/fractionation. Of the seven reported incidents, two were errors in the prescription chosen, with one for a patient not suitable for treatment; the remaining five were related to failures to complete the EAS correctly. Of the two instances of unsuitable prescribed doses/treatment, treatments requested were nonstandard, off‐protocol, and required the involvement of a second clinician who then declined to agree treatment. The key controls for these incidents were the pre‐existence of clearly defined clinical protocols and the requirement for independent clinician review when operating outside of these protocols. Clinical protocols are mandated by UK legislation^6^with peer review recommended^36^ and these measures on these occasions prevented near‐misses from becoming reportable incidents. These incidents also align with a key theme in the local FMEA MDT discussion, namely the limited safety barriers for clinician outlining and prescribing. Deciding on treatment approaches and delineating CTVs are a particularly challenging task^36^ and within the local FMEA existing control measures rely on deviations from the norm being significant enough to be detectable by nonclinician planners or checkers. As the FMEA does not account for a sliding scale of error, the decision at MDT discussion was made to concentrate on the most severe error in outlining which corresponds with a theoretically higher likelihood of detection from nonclinical staff. Reconsidering a failure mode with a more subtle error results in a reduced severity but a significantly poorer chance of detection and a higher likelihood of occurrence. Retrospective rescoring produces a RPN of 240, double the stated RPN score of 120 and an S,O,D code of 658.

The risk of more subtle errors in outlining highlights a key weakness of the current lung radiotherapy planning process at our institute, namely a lack of clinician peer review. Recent guidance from the Royal College of Radiologists^36^ recommends peer review for lung radiotherapy and, if implemented, prospective peer review would significantly reduce the likelihood of error both through standardization of practise and improved detectability from the introduction of a second clinician review as an additional control measure.

### 4.E Resource requirements of FMEA

The resource requirements for conducting an FMEA are under reported in the literature with relatively few papers providing timings.^12^, ^13^, ^15^, ^23^ Reported timings range from 30^23^ to 258^12^ h, dependent on methodology. For a radiotherapy center considering adopting FMEA this information is particularly important, as there is cost associated with embarking on convoluted and lengthy risk assessments, particularly when considering an active service. The example presented here is an attempt at a time limited FMEA, balancing comprehensive risk assessment with practicality. One potential criticism is the use of a single MDT meeting as further meetings may have reduced variance and increased the number of failure modes and/or the granularity of failure modes included. From this experience, future FMEA undertaken locally will include a further meeting to confirm the final analysis. When considering the number of failure modes determined, the total of 40, is lower than previously published values albeit for different specialisms and equipment. As discussed previously, omission of risks associated with commissioning and quality assurance may account for some of this discrepancy as well as the aforementioned limited granularity to individual failure modes. Nevertheless, the work presented here represents FMEA as a feasible option for risk assessment in a busy clinic.

## 5 CONCLUSION

Failure modes and effect analysis can be effectively applied for routine risk assessment of clinical services as required under UK law.^37^ Facilitation can be used to reduce the time burden of the FMEA process to a level manageable for busy departments enabling wider implementation beyond specialist and new services. Comparison with local incident reporting highlights that although an MDT approach can produce a comprehensive list of failure modes, regular comparison with robust local reporting procedures can ensure an inclusive consideration of risk.

## AUTHOR CONTRIBUTION

Martyn Gilmore was the author of the manuscript, providing all written text. Martyn was also the facilitator alluded to in the text and as such provided all preassessment materials, including failure modes and the process map, and carried out all analysis discussed.

Carl Rowbottom was the supervisor of Martyn Gilmore during the work described in the manuscript and carried out reviews of multiple drafts prior to submission. Carl worked with Martyn to develop the structure of work described within.

## CONFLICT OF INTEREST

No Conflict of interest.

## Data Availability

The data that support the findings of this study are available from the corresponding author upon reasonable request.

## References

[1] European Commission . Radiation Protection ‐ General Guidelines on Risk Management in External Beam Radiotherapy. 2015.

[2] Johnston AM . Unintended Overexposure of a Patient during Radiotherapy Treatment at the Edinburgh Cancer Centre, in September 2015. The Scottish Government; 2016.

[3] Briggs G . Towards safer radiotherapy. Natl Patient Saf Agency. Published online. 2008;85.

[4] European Commission . N° 181 General Guidelines on Risk Management in External Beam Radiotherapy; 2015. https://ec.europa.eu/energy/sites/ener/files/documents/RP181web.pdf

[5] American Society for Radiation Oncology A . Safety Is No Accident: A Framework for Quality Radiation Oncology Care. American Society for Radiation Oncology, ASTRO; 2019.

[6] Parliament UK . The Ionising Radiation (Medical Exposure) Regulations 2017 (No. 1322). Stationary Office UK; 2017:1‐24.

[7] International Atomic Energy Agency (IAEA) . Radiation Protection and Safety of Radiation Sources : International Basic Safety Standards General Safety Requirements Part 3; 2014.

[8] Huq MS , Fraass BA , Dunscombe PB , et al. Application of risk analysis methods to radiation therapy quality management: report of AAPM Task Group 100. Med Phys. 2016;43:4209–4262. 27370140 10.1118/1.4947547 PMC4985013

[9] Veronese I , De Martin E , Martinotti AS , et al. Multi‐institutional application of failure mode and effects analysis (FMEA) to cyberknife stereotactic body radiation therapy (SBRT). Radiat Oncol. 2015;10:1–11. 26071401 10.1186/s13014-015-0438-0 PMC4469574

[10] Teixeira FC , de Almeida CE , Saiful HM . Failure mode and effects analysis based risk profile assessment for stereotactic radiosurgery programs at three cancer centers in Brazil. Med Phys. 2016;43:171. 26745909 10.1118/1.4938065

[11] Xu AY , Bhatnagar J , Bednarz G , et al. Failure modes and effects analysis (FMEA) for Gamma Knife radiosurgery. J Appl Clin Med Phys. 2017;18:152–168. 10.1002/acm2.12205 PMC5689925 29082599

[12] Schuller BW , Burns A , Ceilley EA , et al. Failure mode and effects analysis: a community practice perspective. J Appl Clin Med Phys. 2017;18:258–267. 28944980 10.1002/acm2.12190 PMC5689935

[13] Ford EC , Gaudette R , Myers L , et al. Evaluation of safety in a radiation oncology setting using failure mode and effects analysis. Int J Radiat Oncol Biol Phys. 2009;74:852–858. 19409731 10.1016/j.ijrobp.2008.10.038 PMC3655406

[14] Denny DS , Allen DK , Worthington N , Gupta D . The use of failure mode and effect analysis in a radiation oncology setting: the Cancer Treatment Centers of America experience. J Heal Qual. 2014;36:18–28. 10.1111/j.1945-1474.2011.00199.x 22364244

[15] Ford EC , Smith K , Terezakis S , et al. A streamlined failure mode and effects analysis. Med Phys. 2014;41:61709. 10.1118/1.4875687 24877804

[16] Bowles J . An Assessment of RPN prioritization in a failure modes effects and criticality analysis. Proc Annu Reliab Maintainab Symp. Published online. 2003:380–386.

[17] Franklin BD , Shebl NA , Barber N . Failure mode and effects analysis: too little for too much? BMJ Qual Saf. 2012;21:607–611. 10.1136/bmjqs-2011-000723 22447819

[18] Shebl NA , Franklin BD , Barber N . Failure mode and effects analysis outputs: are they valid? BMC Health Serv Res. 2012;12:150. 22682433 10.1186/1472-6963-12-150 PMC3405478

[19] Younge KC , Wang Y , Thompson J , Giovinazzo J , Finlay M , Sankreacha R . Practical implementation of failure mode and effects analysis for safety and efficiency in stereotactic radiosurgery. Radiat Oncol Biol. 2015;91:1003–1008. 10.1016/j.ijrobp.2014.12.033 25670543

[20] López‐Tarjuelo J , Bouché‐Babiloni A , Santos‐Serra A , et al. Failure mode and effect analysis oriented to risk‐reduction interventions in intraoperative electron radiation therapy: the specific impact of patient transportation, automation, and treatment planning availability. Radiother Oncol. 2014;113:283–289. 25465728 10.1016/j.radonc.2014.11.012

[21] Faught JT , Balter PA , Johnson JL , et al. An FMEA evaluation of intensity modulated radiation therapy dose delivery failures at tolerance criteria levels. Med Phys. Published online. 2017;44:5575–5583. 28862765 10.1002/mp.12551 PMC6421844

[22] Ashley L , Armitage G . Failure mode and effects analysis: an empirical comparison of failure mode scoring procedures. J Patient Saf. 2010;6:210–215. 21500607 10.1097/pts.0b013e3181fc98d7

[23] Manger RP , Paxton AB , Pawlicki T , Kim G‐Y . Failure mode and effects analysis and fault tree analysis of surface image guided cranial radiosurgery. Med Phys. 2015;42:2449–2461. 25979038 10.1118/1.4918319

[24] Cancer Research UK . Lung cancer incidence statistics. Published 2017. https://www.cancerresearchuk.org/health‐professional/cancer‐statistics/statistics‐by‐cancer‐type/lung‐cancer/incidence#heading‐Zero Accessed May 10, 2019.

[25] Cancer Survival in England : Patients Diagnosed between 2010 and 2014 and Followed up to 2015; 2016. https://www.ons.gov.uk/releases/cancersurvivalforadultsinengland2015

[26] Otto K . Volumetric modulated arc therapy: IMRT in a single gantry arc. Med Phys. 2008;35:310–317. 18293586 10.1118/1.2818738

[27] National Incident Reporting and Learning System . NRLS Reporting. 2019. https://report.nrls.nhs.uk/nrlsreporting/Default.aspx Accessed May 10, 2019.

[28] Ford EC , De Los F , Santos L , Pawlicki T , Sutlief S , Dunscombe P . Consensus recommendations for incident learning database structures in radiation oncology. Med Phys. 2012;39:7272–7290. 23231278 10.1118/1.4764914

[29] Liu HC , Liu L , Liu N . Risk evaluation approaches in failure mode and effects analysis: a literature review. Expert Syst Appl. 2013;40:828–838.

[30] Masini L , Donis L , Loi G , et al. Application of failure mode and effects analysis to intracranial stereotactic radiation surgery by linear accelerator. PRRO. 2014;4:392–397. 10.1016/j.prro.2014.01.006 25407860

[31] NHS Improvement . Never Events policy and framework 2018. 2018;(January):1‐13. https://improvement.nhs.uk/documents/2265/Revised_Never_Events_policy_and_framework_FINAL.pdf

[32] Care D of H and S . Guidance to the Ionising Radiation (Medical Exposure) Regulations 2017; 2018. https://assets.publishing.service.gov.uk/government/uploads/system/uploads/attachment_data/file/720282/guidance‐to‐the‐ionising‐radiation‐medical‐exposure‐regulations‐2017.pdf

[33] Carthey J . Clinical Human Factors Group, Implementing Human Factors in healthcare ‘How to’ guide ‐ volume 2 ‘Taking further steps.’ Published online 2013. www.chfg.org

[34] Yang F , Cao N , Young L , et al. Validating FMEA output against incident learning data : a study in stereotactic body radiation therapy. Am Assoc Phys Med. 2015;42. 10.1118/1.4919440 26127030

[35] Ford EC , Evans SB . Incident learning in radiation oncology: a review. Med Phys. 2018;45:e100–e119. 29419944 10.1002/mp.12800

[36] The Royal College of Radiologists . Radiotherapy target volume definition and peer review RCR guidance. *R Coll Radiol*. Published online 2017. https://www.rcr.ac.uk/publication/radiotherapy‐target‐volume‐definition‐and‐peer‐review 10.1016/j.clon.2019.07.021 31444023

[37] Department of Health . Guidance on investigation and notification medical exposures much greater than intended. 2017;4(5):1‐6. https://www.gov.uk/government/uploads/system/uploads/attachment_data/file/583637/MGTI_guidance_Jan_17.pdf

